# Assisted Suicide in Parkinsonian Disorders

**DOI:** 10.3389/fneur.2021.656599

**Published:** 2021-03-15

**Authors:** Georg S. Nuebling, Elisabeth Butzhammer, Stefan Lorenzl

**Affiliations:** ^1^Department of Neurology, Klinikum der Universität München, Ludwig-Maximilians-University, Munich, Germany; ^2^Department for Palliative Medicine, Klinikum der Universität München, Ludwig-Maximilians-University, Munich, Germany; ^3^Professorship for Palliative Care, Institute of Nursing Science and –Practice, Paracelsus Medical University, Salzburg, Austria; ^4^Department of Neurology, Klinikum Agatharied, Hausham, Germany

**Keywords:** assisted suicide [MeSH], progressive supranuclear palsy, multiple systems atrophy, corticobasal syndrome, Parkinson's disease

## Abstract

**Background:** Due to the high prevalence of suicidal ideation in Parkinson's Disease (PD) and exploratory data indicating a similar prevalence in atypical Parkinsonian disorders (APD), we sought to determine the frequency of assisted suicide (AS) as well as factors driving these decisions in PD and APD.

**Methods:** Retrospective chart analysis (2006-2012) at a Swiss Right-to-Die organization. Patients with PD and APD who completed AS were analyzed concerning disease state, symptom burden, medication, and social factors.

**Results:** We identified 72 patients (PD = 34, PSP = 17, MSA = 17, CBS = 4; 7.2% of all AS cases), originating mainly from Germany (41.7%), Great Britain (29.2%), and the US (8.3%). Predominant symptoms at the time of application were immobility (PD/APD: 91%/97%), helplessness (63%/70%), pain (69%/19%), dysarthria (25%/32%), and dysphagia (19%/59%). APD patients generally showed a higher symptom burden and a higher frequency of diagnosed depression (8.8%/28.9%). While most patients with diagnosed depression received antidepressants (80%), other symptoms such as pain (59%) were treated less consistently. Of note, time from diagnosis to application differed greatly between PD (8.5 ± 6.8 years) and APD (1.5 ± 1.3 years, *p* < 0.0001).

**Conclusions:** In our analysis, Parkinsonian disorders appeared to be overrepresented as a cause of AS considering the prevalence of these diseases. The observation that assisted suicide is sought early after initial diagnosis in APD implies the need for early comprehensive psychological support of these patients and their relatives.

## Introduction

Parkinson's disease (PD) and atypical Parkinsonian disorders (APD) such as Progressive Supranuclear Palsy (PSP), Multiple Systems Atrophy (MSA), or Corticobasal Syndrome (CBS) impose an immense burden on patients and caregivers. Given the severely limited life expectancy and rapid symptom progression, these diagnoses may lead to existential crises especially in APD, where treatment options are limited to symptom control and based mostly on retrospective case series and anecdotal evidence (1). While single case reports describing assisted suicide (AS) in PD and APD have been published, little is known about the frequency and circumstances of AS in parkinsonian disorders.

Suicidality in general has been extensively studied in PD, especially in the context of deep brain stimulation (DBS) (2). It was discovered that suicidality may be increased during the first year after DBS surgery (3). In contrast, suicide frequencies in the overall PD population appeared not to differ greatly from the general population, although the results of various studies are inconsistent (4–6). Conversely, it was demonstrated that suicidal ideation is highly prevalent in PD (~30%) (6, 7). In addition, a high frequency of depression was determined in PD patients with a reported prevalence of up to 58% (8, 9).

In contrast, suicidality and especially AS in APD has not been the target of many studies, despite single published cases drawing attention toward this topic (10, 11). In MSA, a prevalence of suicidal ideation of 18.4% was described in a cross-sectional study exploring neuropsychiatric symptoms in 48 patients. A Chinese study exploring causes of death in 138 MSA patients determined a suicide rate of 2.8%. We recently noted a high frequency of suicidality (19%) and death ideation (16%) in a small cohort of PSP patients (*n* = 31) (12). Of note, the authors of one case report describing a patient diagnosed with probable PSP who committed suicide concluded that his course of action was likely due to increased impulsivity rather than depression (13). Furthermore, a large cross-sectional study on forensic autopsies in Japan identified clinically undiagnosed cases of PSP (2.9% of cases) (14). Strikingly, a high number of these cases (37.9%) had committed suicide, although the circumstances of these suicides have not been explored further.

Currently, the legal situation concerning AS is heterogeneous across Europe and a subject of ongoing debate, with physician assisted suicide being legally permitted in Belgium, Luxembourg and the Netherlands, and non-medical Right-to-Die organizations guiding assisted suicide in Switzerland. While the intention of assisted suicide facing a severely disabling and life-limiting condition is accepted as a final act of autonomy in these countries, the situation is complex in the case of parkinsonian disorders. Importantly, concomitant depression is highly frequent in these diseases, and may sometimes be hard to diagnose or treat. Moreover, frontal disinhibition and symptoms of dementia may undermine a patient's ability for informed consent. Lastly, options for symptomatic (pharmacological and non-pharmacological) treatment as well as patient and family support may not be available in certain areas.

Given the lack of knowledge concerning the circumstances of assisted suicide in parkinsonian disorders, we retrospectively analyzed clinical data from a Swiss Right-to-Die organization to determine the number of patients with PD or APD who completed assisted suicide as well as factors potentially driving these decisions. The organization's archives comprised detailed medical and sociodemographic data acquired during the application process. In brief, patients applying for AS are required to undergo two clinical interviews conducted by different physicians, comprising a detailed medical history, physical examination and exclusion of conditions that might interfere with the applicant's ability to consent. Furthermore, current and past medical reports have to be provided. In this process, detailed information concerning alternative treatment options including palliative care is provided by the physicians.

## Materials and Methods

### Retrospective Data Acquisition

The retrospective analysis was conducted at the archive of a Swiss Right-to-Die organization, where unrestricted access was provided to SL and EB. Patients with a clinical diagnosis of PD or APD (PSP, MSA, CBS) who underwent assisted suicide in the years 2006-2012 were identified. Patients with a diagnosis of PD or APD who sought out the organization due to other comorbidities (e.g., malignancies) were excluded. Clinical data was extracted from the archives and anonymized upon extraction. Extracted data included basic demographic information, prior diagnoses, medication, symptom burden as well as circumstances of the application and administration process. If not documented, Hoehn and Yahr scales were retrospectively determined from documented physical examinations.

### Statistical Analyses

Numerical data was controlled for normal distribution by D'Agostino and Pearson test, homogeneity of variance was determined by Levene's test. Comparisons of numerical data between APD and PD patients were done by Mann-Whitney *U*-test due to non-normality. Binominal data was compared applying Fisher's exact test. Correction for multiple comparisons was not done given the exploratory nature of the study. *P* < 0.05 was considered statistically significant.

### Ethical Considerations

This study was approved by a local institutional review board (application number 17-090) and was conducted in accordance with the Declaration of Helsinki in its most recent revision.

## Results

### Study Population

We identified 72 patients with a primary diagnosis of PD (*n* = 34), PSP (*n* = 17), CBS (*n* = 4), or MSA (*n* = 17) who committed assisted suicide. These patients made up 7.2% of cases in the investigated time period. Demographic data are summarized in Table 1. Countries of origin were Germany (41.7%), Great Britain (29.2%), the United States of America (8.3%), France (5.6%), Canada (4.2%), Switzerland/Spain/Austria (each 2.8%), and Portugal/Czech Republic (each 1.4%). Concerning marital status, 51.4% of the study population were married or in a relationship. 18.1% resided in a nursing home, and the majority of patients (91.4%) was accompanied by family or friends during the assisted suicide. 59.3% (32/54) of the patients with available documentation concerning religious affiliation were members of a Christian denomination, whereas no other religious affiliations could be identified.

**Table 1:** Demographic analysis, symptoms, and pharmaceutical treatment of the *AS* cohort.

	**PSP**	**MSA**	**CBS**	**APD**	**PD**	***p*-value APD vs. PD**
**Demographic data**						
***n*** **(%)**	17 (23.6%)	17 (23.6%)	4 (5.6%)	38 (52.8%)	34 (47.2%)	
Female gender; *n* (%)	9 (52.9%)	10 (58.8%)	2 (50.0%)	21 (55.3%)	15 (44.1%)	0.479
Age (years); mean(SD)	68.0 (6.4)	64.4 (8.8)	60.7 (11.6)	65.6 (8.2)	72.4 (12.5)	**0.0035**
Married/in relationship; *n* (%)				23 (60.5%)	14 (41.2%)	0.156
Patients without offspring; *n* (%)	4 (13.5%)	5 (29.4%)	1 (25.0%)	10 (26.3%)	13 (38.2%)	0.319
Nursing home resident; *n* (%)	2 (11.8%)	5 (29.4%)	0 (0%)	7 (18.4%)	6 (17.6%)	0.999
Member of a religion; *n* (%)	6 (54.5%) *n* = 11	6 (46.2%) *n* = 13	4 (100%)	16 (57.1%) *n* = 28	16 (61.5%) *n* = 26	0.787
Disease duration (years); mean(SD)	4.7 (1.6)	5.5 (4.4)	4.2 (2.0) *n* = 3	5.5 (3.2) *n* = 37	11.6 (6.9) *n* = 21	**0.00023**
Hoehn and Yahr stage; mean(SD)	4.6 (0.5) *n* = 16	4.3 (0.8)	3.75 (1.5)	4.4 (0.8) *n* = 37	4.1 (1.0) *n* = 33	0.210
Diagnosed depression; *n* (%)	4 (23.5%)	5 (29.4%)	2 (50.0%)	11 (28.9%)	3 (8.8%)	**0.039**
Previous suicide attempt; *n* (%)	3 (17.6%)	2 (11.8%)	1 (25.0%)	6 (15.8%)	4 (11.7%)	0.740
Time from diagnosis to AS application (years); mean(SD)	1.2 (0.9)	2.0 (1.8)	1.3 (0.2)	1.5 (1.3)	8.8 (6.7) *n* = 23	**<0.0001**
Time from application to AS (days); median(range)	127 (21–452)	115 (39–422)	60 (31–94)	105 (21–452)	99 (6–2090) *n* = 23	0.970
Patients accompanied by family and/or friends; *n* (%)	16 (94.1%)	13 (86.7%) *n* = 15	4 (100%)	33 (89,2%) *n* = 37	31 (93.9%) *n* = 33	0.677
**Symptoms**						
***n*** **(%)**	17 (24.6%)	16 (23.2%)	4 (5.8%)	37 (53.6%)	32 (46.4%)	
Helplessness	11 (64.7%)	11 (68.8%)	4 (100%)	26 (70.3%)	20 (62.5%)	0.610
Immobility	17 (100%)	15 (93.8%)	4 (100%)	36 (97.3%)	29 (90.6%)	0.330
Dysarthria	17 (100%)	13 (81.3%)	2 (50.0)	32 (86.5%)	8 (25.0%)	**<0.0001**
Dysphagia	13 (76.5%)	8 (50.0%)	1 (25.0%)	22 (59.5%)	6 (18.8%)	**0.0007**
Impaired vision	10 (58.8%)	2 (12.5%)	1 (25.0%)	13 (35.1%)	7 (21.9%)	0.291
Pain	6 (35.3%)	10 (62.5%)	1 (25.0%)	17 (45.9%)	22 (68.8%)	0.0878
Urinary incontinence	1 (5.9%)	9 (56.3%)	1 (25.0%)	11 (29.7%)	6 (18.8%)	0.403
**Medication/treatment**						
***n*** **(%)** (patients with available medication)	13 (76.5%)	10 (58.8%)	2 (50.0%)	25 (65.8%)	27 (79.4%)	
L-dopa	3 (23.1%)	4 (40.0%)	0 (0%)	7 (28.0%)	24 (88.9%)	**<0.0001**
Dopamine-agonists	1 (7.7%)	2 (20.0%)	0 (0%)	2 (8%)	8 (29.6%)	0.077
COMT-inhibitors	1 (7.7%)	0 (0%)	0 (0%)	1 (4.0%)	7 (25.9%)	0.051
MAO-B-inhibitors	0 (0%)	0 (0%)	0 (0%)	0 (0%)	2 (7.4%)	0.491
Amantadine	5 (38.5%)	1 (10.0%)	0 (0%)	6 (24.0%)	6 (22.2%)	0.999
Deep brain stimulation	0 (0%)	0 (0%)	0 (0%)	0 (0%)	1 (3.7%)	0.999
Antidepressants	10 (76.9%)	2 (20.0%)	2 (100%)	14 (56.0%)	6 (22.2%)	**0.0217**
Neuroleptics	0 (0%)	0 (0%)	0 (0%)	0 (0%)	5 (18.5)	0.0515
Benzodiazepines	4 (30.8%)	2 (20.0%)	2 (100%)	8 (32.0%)	2 (7.4%)	**0.0356**
ACh-esterase inhibitors	0 (0%)	0 (0%)	1 (50.0%)	1 (4.0%)	2 (7.4%)	0.999
Prokinetics	1 (7.7%)	1 (10.0%)	0 (0%)	4 (16.0%)	3 (11.1%)	0.698
Cannabinoids	0 (0%)	0 (0%)	0 (0%)	0 (0%)	1 (3.7%)	0.999
Analgetics	2 (15.4%)	6 (60.0%)	1 (50.0%)	10 (40.0%)	9 (33.3%)	0.999

*Bold values in the right column highlight significant p-values*.

### Comparison of PD and APD Patients

**Figure 1:**
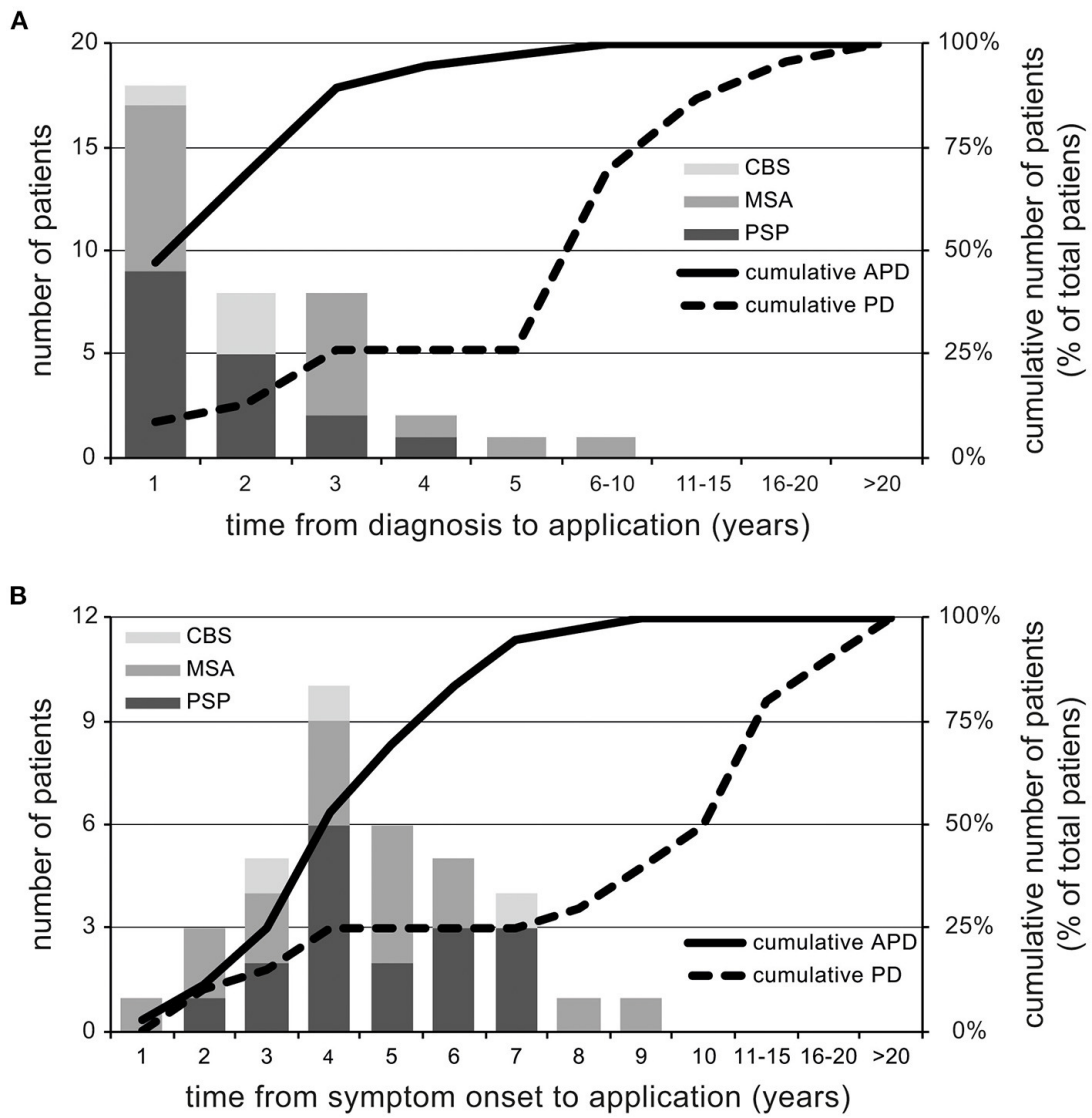
Time to application for assisted suicide. Depiction of the annual application rate for assisted suicide from the time of diagnosis **(A)** and symptom onset **(B)** as well as cumulated percentages of applications for both PD and APD patients. Of note, time from diagnosis to application is markedly reduced in APD as compared to PD.

Compared to PD, APD patients were overrepresented in this study population (*n* = 38; 52.8%) considering the overall lower prevalence of PSP, MSA, and CBS. APD patients were younger and had significantly lower disease durations as compared to PD (see Table 1). No differences in gender, marital status, total disability as measured by Hoehn and Yahr scale and nursing home residency was observed (see Table 1). Of note, APD patients had a higher frequency of diagnosed depression. The most striking difference to PD patients was a much lower time from diagnosis to application for assisted suicide especially in PSP and CBS. In APD, assisted suicide was applied for within the first year after diagnosis by 47.4% (PSP 52.3%, MSA 47.1%, CBS 25%) of all patients, whereas this was observed in only 8.7% of PD cases (see Figure 1).

### Symptom Burden and Medication

APD patients generally exhibited a tendency toward higher symptom burden in all disease-associated symptoms except for pain, reaching statistical significance for dysarthria and dysphagia (see Table 1). Concerning treatment, it is noteworthy that a high number of patients who had pain documented as a relevant symptom did not receive any pain medication [PD: 75% (15/20); APD: 33% (4/12)]. In contrast, 80% of patients with a diagnosis of depression among those whose prior medication was available had received antidepressants. L-Dopa was used more frequently in PD, whereas more APD patients were prescribed antidepressants and benzodiazepines (see Table 1). One PD patient had received deep brain stimulation.

## Discussion

Patients suffering from parkinsonian disorders face a wide variety of disabling symptoms. Especially in APD, delays in the diagnostic process and limited knowledge about disease trajectories impose a massive burden on patients and caregivers alike. Once a diagnosis of APD is made, patients have to cope with limited symptomatic treatment options. Although promising novel curative therapeutic strategies such as targeting cerebral deposits of aggregated proteins are currently developed, access to treatment trials is limited, and outcomes are unknown. Hence, applying for assisted suicide may be considered as a last resort of autonomous choice by some patients.

In our study, parkinsonian disorders made up a relevant fraction of total AS applications (7.2%) considering their contribution to overall mortality. In general, neurological disorders make up a relevant fraction of AS cases in Switzerland, with reported neurological diagnoses varying from ~12 to ~47% (15). The broad range of reported neurological diagnoses may in part be explained by the methodology applied, with some studies reporting only the primary diagnosis, whereas others allowed for multiple diagnoses. Some, but not all, studies have reported an increase in the fraction of neurological disorders over time (15, 16). APD cases appeared to be overrepresented as compared to PD patients in this study. Importantly, the time from diagnosis to application for assisted suicide was very short in APD as compared to PD patients. This implies a need for a stronger support network especially for newly diagnosed APD patients.

From our findings, several factors potentially driving a decision for assisted suicide can be discussed. Depression is a factor repeatedly identified as potentially influencing death ideation, with a high prevalence in parkinsonian disorders, possibly highest in PSP and CBS (8, 9, 17, 18). Depression was also highly prevalent in the APD population studied here, where a previous diagnosis of depression was documented in 28.9% of cases, despite the fact that the condition can be difficult to identify in the APD population. Another notable factor identified in our current analysis was a potential discrepancy between symptom burden and medication. A high number of PD patients and (to a lesser extent) APD patients reported pain upon presentation. However, pain medication was documented for only a fraction of these patients. It could thus be argued that insufficient symptom control may be a potential contributor in the decision making process.

Concerning social factors, it was noted that most patients committing assisted suicide were accompanied by family or friends, and less than one fifth of AS cases had lived in a nursing home. In a previous exploratory study, partnership status was not associated with suicidality in PSP (12). However, we have already shown that relatives living with these patients have a high rate of depression, which might influence the patient's decision (19). It is thus possible that the motivation to apply for AS may at least in part originate from a desire not to impose additional burden on spouses and caregivers. Such motives were also noted in another small German cohort (20). These findings may further be explained by the limited access to AS of immobilized patients who do not receive support by spouses or friends, e.g., residents of nursing homes, as it was shown that family members often take on an important role in supporting the decision for AS as well as the organizational process (21).

The current study has several limitations. First, data on patients who committed assisted suicide was collected retrospectively and may thus be incomplete, and correct diagnoses could not be verified by the investigators. This has to be taken into account when drawing conclusions from documented symptoms and medication, and especially when looking at Hoehn and Yahr stages, since these often had to be reconstructed from documented physical examinations. However, we noted that especially documentation of symptom burden was very detailed (both self-reported and caregiver-reported), as was required to prove the patient's high degree of suffering. Furthermore, it has to be considered that in this uniquely vulnerable patient collective, a prospective analysis as the method of choice would face intricate ethical challenges. Second, the investigation was limited to only one of the several Swiss organizations providing AS given its exploratory nature. The observation that almost no Swiss patients applied at the organization most likely reflects the fact that this organization is one of the few providing services to non-Swiss nationals. Thus, the results presented here rather reflect the situation of AS applicants from European countries where AS is not permitted, but not necessarily that of Swiss patients.

In summary, the data presented here prompt physicians to proactively assess a potential need for psychological support after an APD diagnosis is made, and to actively address the wish to hasten death. Thorough assessment and consequent treatment of both motor and non-motor symptoms seems warranted.

## Data Availability Statement

The datasets presented in this article are not readily available because this would jeopardize patient confidentiality. Requests to access the anonymized datasets should be directed to stefan.lorenzl@pmu.ac.at.

## Ethics Statement

The studies involving human participants were reviewed and approved by Ethikkommission bei der LMU München. Written informed consent for participation was not required for this study in accordance with the national legislation and the institutional requirements.

## Author Contributions

GN: study conception, statistical analysis (design, execution), manuscript draft, and approval of the final manuscript. EB: study execution and organization, statistical analysis (design, critical review), critical review of the manuscript, and approval of the final manuscript. SL: study conception and organization, statistical analysis (critical review), critical review of the manuscript, and approval of the final manuscript. All authors contributed to the article and approved the submitted version.

## Conflict of Interest

The authors declare that the research was conducted in the absence of any commercial or financial relationships that could be construed as a potential conflict of interest. The handling editor declared a past collaboration with one of the authors SL.
